# Uterine Displacements

**Published:** 1868-02-01

**Authors:** L. A. Babcock

**Affiliations:** Freeport, Ill.


### UTERINE DISPLACEMENTS.

BY L. A. BABCOCK, M.D., FREEPORT, ILL.

DISPLACEMENTS of the Uterus are of three kinds, viz.: PROLAPSUS, RETROVERSION, and ANTEVERSION. The uterus occupies normally very nearly a central position in the pelvis, being, perhaps, a little nearer to the sacrum than to the pubis, its long axis should stand at about right angles to that of the vagina, the fundus pointing in the direction of the umbilicus, and the os tincæ towards the end of the coccyx. Prolapsus signifies a sinking of the uterus below its normal position, and sometimes it sinks nearly or quite down to the os externum; when it protrudes beyond the vulva, it is called procidentia uteri. The fundus may be tilted a little one way or the other without the position being necessarily abnormal. The condition and contents of the bladder and rectum may temporarily influence it to some extent.

If it turns forwards or backwards for 25 degrees or 30 degrees, it does not amount to malposition, but if to 40 degrees in either direction, without soon rectifying itself, it is abnormal, and usually goes from bad to worse, till the malposition becomes persistent. If the uterus fall backward in a line drawn from the os to the promontory of the sacrum, it will describe an angle of 45 degrees, and will present its broadest surface to the pressure of the superincumbent viscera, which will necessarily force it eventually lower and lower; and if it turned forward to the same extent, the same power exerted on its broad posterior surface, necessarily increases this abnormal tendency, But an anteversion never goes relatively to so great an extent as a retroversion, simply because it meets with more resistance. Anteversion often stops at 45 degrees, but may go to 90 degrees, as when we have a complete version, with the whole organ lying flatly down on the entire wall of the vagina, and parallel with it, while a retroversion seldom or never stops under 90 degrees, and often goes to 135 degrees, simply because there is less opposition to its downward progress. It then follows that if the fundus of the uterus is found constantly lying just behind or even near the symphysis pubis, it is an anteversion, but if it is found lying persistently back under the promontory of the sacrum, it is a retroversion.

Some practitioners are skeptical as to the injurious consequences which result from these several displacements of the uterus. The patients complain both of local pain and constitutional suffering, while they obstinately refuse to be comforted by the assertion that it is all "hysteria." But an experience of thirty years in the practice of medicine has convinced me that a displacement of the uterus, is just as much an abnormal occurrence as is the dislocation of a joint.

To say that the pain and other symptoms which accompany uterine displacements are simply due to congestion or inflammation, must lead to a course of treatment as absurd and mischievous as if a dislocated humerus or femur were to be treated by leechings and alterative drugs without attempting reduction.

If the profession would turn to be moral reformers, and try to correct some of the great evils of society, they might lecture to women on their bad habit of tight lacing, which tends to force the bowels down into the pelvic cavity, and is a very prolific source of prolapsus. But another and more degrading custom is the habitual use of periodical pills and drugs, to prevent conception, which, more than anything else I know of, tends to produce displacement and leucorrhœa.

But the profession have to deal with facts as they exist. We have to diagnose a case from its history and present condition, without looking back to our grandmothers to see what diseases they may, or may not have had, in a former age of prudence, purity, and virtue.

To treat successfully these several displacements, surgical appliances must be adapted for rectifying the malpositions of the womb, and for its retention as nearly as possible in its normal position.

The womb must be restored to its normal position in the pelvis, and then it must be held there by an instrument that does not irritate the cervex or vagina, nor rest upon the perineum for its support. And the instrument must be small and light, and of a non-irritating substance, of silver or gold, so that its galvanic power will tone and strengthen the enervated uterine organs. Astringent injections do no good, and caustics decided harm. And as to rubber, and glass pessaries — either globe, horseshoe, or any other shape — when they are to be crammed into the vagina, with no base external to support them, must, of necessity, increase the irritation and make the condition worse.

A surgeon might as well cram a load of hay into the vagina to support the uterus in prolapsus as a rubber pessary, and with as good a chance of success, for both would cause irritation and leucorrhœa, and to blow up a wind bag in the vagina is more ridiculous.

What can be more humiliating to the student than to look over the different shaped instruments that have been used in the treatment of uterine displacements, and yet, teachers of medicine still urge the use of these worthless rubber and glass pessaries that expand the vagina, and prevent its contractions to support the uterus, resting upon the perineum for its support, causing constipation and leucorrhœa, and a train of evils worse than displacement itself. And even when a stem pessary is used, many still cling to the old fogy idea of a rubber with a harness as appropriate as an ox yoke would be, or a hay rack, with all its projecting points, or still worse, a stem pessary "elevator," supported by straps that close up the anus and urethra, so that it has to be taken off whenever there is a movement of the bowels, or micturition, the very time the support is most needed.

The instrument I use consists of a silver cup, made to nicely fit the neck of the womb, attached to a hollow curved silver stem, which fits closely to a silver handle, dividing in a curve over the vulva and os pubis, and is fastened and held firmly in its place by a belt around the body above the pelvis. When this instrument is properly adjusted, the patient can walk, sit, or work with perfect ease, as it does not touch the bowels, nor urethra; micturition is not impeded, nor is the vagina expanded or irritated, for there is not a joint, rough or uneven place in the instrument, it all being polished silver, it will not corrode, and is so light that it is worn with comfort. And the silver itself tends to tone the organ, and is the only metal that can properly be used in such instruments.

Any physician who wishes to try one of these instruments, can have one at the actual cost of manufacture, by addressing me as per above.

